# Innovation in the 21st century: following the footsteps of Katalin Karikó

**DOI:** 10.1007/s42977-023-00161-8

**Published:** 2023-05-22

**Authors:** Csaba Deák, Norbert Pardi, Ádám Miklósi

**Affiliations:** 1 Faculty of Materials and Chemical Engineering, University of Miskolc, Miskolc, Hungary; 2 Department of Microbiology, Perelman School of Medicine, University of Pennsylvania, Philadelphia, PA USA; 3 ELKH-ELTE Comparative Ethology Research Group, Budapest, Hungary; 4 Department of Ethology, Eötvös Loránd University, Budapest, Hungary

**Keywords:** mRNA vaccine, Katalin Karikó, Nucleoside-modification, Innovation, COVID-19, Evolution

## Abstract

Innovation is a critical component of human society, setting us apart from other animals. We possess a unique capacity to design and produce new things through cultivating a culture that values and encourages innovation. One remarkable instance of innovation in the field of biology and medicine is the mRNA vaccine platform developed by Katalin Karikó and her colleagues. In this article, we delve into the evolution of mRNA-based therapy, beginning with animal models and concluding with the first clinical trials. The history of mRNA research began with the identification of its role in protein synthesis, leading to the development of mRNA vaccine technology. Karikó's pivotal innovation was discovering the need to integrate modified nucleosides into the mRNA, decreasing its recognition by the immune system. Her story offers valuable lessons, including the importance of market demand as a booster effect, the role of emerging technologies, the significance of universities and academic institutions in fostering innovation, the role of perseverance and faith, and the role of chance.

## Innovation: taking a long perspective

Innovation, inventing novel behavioural solutions in a population, is older than humanity (Reader and Laland 2003). We do not know who were the first innovators on Earth but the evolutionary importance of innovation was already apparent in the 1950s, as ethologists observed the behaviour of a small group of Japanese monkeys (*Macaca fuscata*) on Koshima island (Hirata et al. 2001). They wanted to learn more about the monkey’s social structure, so to keep the animals interacting within the view of the researchers; they started to provide them with sweet potatoes close to the seashore. One day the researchers noticed that one young, 1.5-year-old female monkey (who was named Imo) picked up a potato, ran into the shallow water, and immersed the food into the water while rotating it. For a human observer, this looked like ‘washing off’ the sand from the potato. The first observation occurred in September 1953. The mother of Imo and another unrelated young male adopted this habit in the same year, but only one young monkey was displaying this behaviour in 1954. One year later, three young monkeys joined the club of potato washers. The spread of the habit of potato washing was very slow. In 1962 (8 years after the discovery of Imo), 74% of the monkey population washed the potatoes. The main reason for this slow transition was that only the younger monkeys (between 1 and 7 years) were inclined to adopt this behaviour. It is an interesting addition, that many years later, in 1956, Imo was again an innovator because instead of picking up wheat grains from the sand, she carried a handful sand with grains to the water and released the load. The sand sank to the bottom and the grains floated on the water surtace. Hundreds of similar observations in many animal species show that the innovative behaviour is wider spread than it is thought (Ramsey et al. 2007).

Studying innovation in animals has shed light on several critical factors that contribute to the emergence of this behaviour, devoid of complex human factors. Innovations may depend on individual characteristics such as curiosity, neophilia, behavioural flexibility, and playfulness, among others. Additionally, the ecological niche and social environment may or may not provide challenges, tools, and other means to arrive at novel solutions. Finally, there is a need for time to explore the problem and interest in learning through observation or teaching. While various animal species possess some combination of these traits, they typically exhibit no strong inclination to evolve an innovative culture.

## Human innovation

According to Reader and Laland (2003), “*innovation is a new modified learnt behaviour not previously found in a population*” (as a product) or “*it is a process that results in new or modified behaviour and that introduces novel behavioural variants into the population’s repertoire*”. There are myriad definitions for innovation in the social and industrial sciences, for example Damanpour and Schneider (2009) refers to innovation as “*the development (generation) and/or use (adaption) of new ideas or behaviours*” and products (our addition).

The European Inventor Award was established in 2006 to reward researchers for their strive to expand the possibilities for humanity: *“The driving force behind the innovation process is people—people with a passion for discovery. Without their inquisitive minds, their quest for new ideas and their creativity, there would be no inventive spirit and no progress”*. Especially in the western culture, there is an emphasis on the role of the inventor in the innovations. Laws have been introduced to protect the inventors’ rights by providing them with advantages over the use of their invention.

**Fig. 1:**
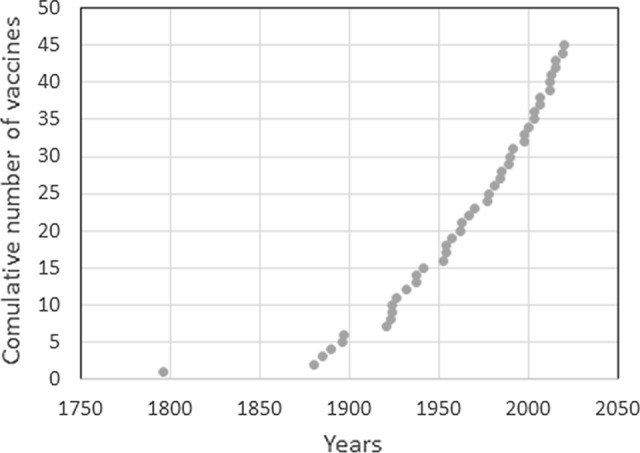
The cumulative history of vaccination. The speed of developing new vaccines increased parallel to the accumulating knowledge about bacteria and viruses

One could also take a broader view to understand why humans are so radically different from non-humans. Part of the story could be that animals are not inventive per se, or their lifestyle and ecology constrains the emergence of such skills. But, there are probably additional factors involved. Tomasello (1999) was the first who applied the metaphor of a ratchet to explain the cultural evolution in humans. The possibility of actively sharing (by language and teaching) individual innovations among group members and with the society not only speeds up the spread of the innovation but also provides a new environment for the manifestation of new innovations which further develop the original idea. This feature seems to be specific to humans among primates, and it has become more pronounced in the exponentially growing modern human population. An early form of vaccination was already practiced in ancient China for centuries, yet the rate of related innovations increased significantly once a mechanistic explanation between infection and disease was established, and a link between prior vaccination and decreased disease severity was observed (Fig. 1). This suggests that culture plays a significant role in the tendency to innovate, and innovations can, in turn, change culture.

In the following section, we provide a brief overview of the development of the mRNA vaccine platform, which played a crucial role in mitigating the severe consequences of the COVID-19 pandemic. This technology has the potential to revolutionize how we prevent and treat many infectious and non-infectious diseases. While many factors contributed to the successful implementation of the mRNA vaccine concept against COVID-19, we focus here on the contribution of mRNA science, from the discovery of the molecule to its transformation into a medical product.

## The mRNA story: first steps

As soon as DNA’s central role in heredity became clear, it has been hypothesized that there must be a specific molecule that provides a connection between the DNA located in the cell nucleus and the ribosomes in the cytoplasm, which are responsible for protein production. As an effort of many eminent scientists, for example, Sydney Brenner, Francois Jacob, Mat Meselson, Charles Kurland, Robert Risebrough, Francis Crick and James Watson, the existence of the molecule was supported, and based on its function, this type of RNA was given the prefix “messenger” (Cobb 2015).

Later research clarified the details of mRNA’s involvement in protein synthesis and also revealed molecular mechanisms that can control gene expression programmes through the regulation of mRNA synthesis (e.g. splicing) and degradation (e.g. poly(A) tails).

### Common knowledge on the use of mRNA in medical treatments

The discovery of the universality of the “central dogma” (the transcription of the genetic information from DNA to mRNA and then its translation to proteins) fascinated biologists over the world. Molecular genetics and biochemistry seemed to offer a radically new way of treating diseases. An air of general optimism resulted in substantial amounts of public and private money flowing into molecular research, which drove the field forward at a speed that was unimaginable before.

Although there was initial optimism for the direct development of mRNA-based treatments, the complexities of the posttranscriptional modifications and intricacies of the translational process, which were both unknown and/or underappreciated at the time, meant that these treatments were bound to fail initially (as noted by Karikó et al. 2008). Nonetheless, there were compelling reasons to favour mRNA over (plasmid) DNA, recombinant protein, and viral vectors:

### Simpler and faster

1. mRNA provides a rapid mechanism of action: because of its efficient transfection of primary cells, it can directly capitalise the translational machinery of the target cells;
2. mRNA is translated rapidly, within minutes after entry into the cytoplasm;
3. mRNA offers a better control of the duration and extent of protein production because it has a shorter and controllable half-life compared to DNA;
4. mRNA has a different molecular structure from DNA; thus, it cannot integrate into the genome posing less threat of deleterious side effects;

### Practical advantages

(5) The mRNA can encode larger protein molecules because the translation process takes place inside the host cell;
(6) The manufacturing of mRNA is simpler than the production of recombinant proteins because the process is scalable and sequence-independent and no difficult purification steps are included;

Obviously, the use of mRNA-based therapies also posed a particular set of new problems:

1. The mRNA molecules are known to stimulate the immune system that results in inflammatory responses;
2. The synthesis of man-made mRNA molecules was not solved until the 1980s;
3. Typical mRNAs degrade too fast, making their clinical application very doubtful;
4. The lack of safe and effective molecules for the in vivo delivery of mRNA posed a critical hurdle until the 2010s.

Despite the aforementioned advantages, these problems pose significant challenges to researchers. The situation seemed unsolvable and ultimately many researchers started to look for other possibilities. Even twenty years later, one of Karikó’s grant proposal was rejected on the ground that “*mRNA is not suitable for therapy, as it is immediately degraded*” (Katalin Karikó personal communication 2022).

## From the use animal models to the clinical trial

In one of the first major developments for the mRNA-field, Melton et al. (1984) and Melton (1985) developed a method for the *in vitro* synthesis of single stranded RNAs of “*virtually any structure”*. This methodological breakthrough also made possible to test anti-sense approaches: when injected a specific anti-sense mRNA was found to be effective in blocking the natural translation of the corresponding protein. A few similar experiments were performed in other laboratories; interestingly, the research group in which Katalin Karikó was working also reported a transient effect of foreign DNA transfection (by the means of liposome capsules) on cultured mammalian cells. The presence of the foreign DNA could be detected for a few days (Somlyai et al. 1985).

Malone et al. (1989) found an efficient method to deliver various amounts of mRNA encapsulated in cationic lipid into cultured murine cells. The activity of the transcribed protein showed positive correlation with the transfected mRNA. They suggested that the method provided an efficient way to transfect cells of any species with mRNA. As a next step, Wolff et al. (1990)* injected reporter protein-encoding mRNAs into* mouse skeletal muscle *in vivo* and demonstrated protein production from the mRNAs.

*About two years after* the first *in vitro* synthesis of single stranded RNAs, Jirikowski et al. (1992) succeeded in reversing diabetes insipidus in a rat model by injecting vasopressin-encoding mRNA into the lateral hypothalamus. The expressed protein acted similarly to the endogenous one by raising urine osmolarity. In another study, mice treated with repeated doses of human CEA (carcinoembryonic antigen), administered intramuscularly, developed anti-carcinoembryonic antibodies (Conry et al. 1995), in contrast to controls.

This line of research led to the first phase 1 clinical trial, in which patients with metastatic prostate cancer were administered prostate specific antigen (PSA) mRNA-transfected dendritic cells. Heiser et al. (2002) found that the vaccine showed bioactivity by inducing PSA-specific immunity and proved to be safe for the patients. A commentary on this therapy acknowledged the significance of this achievement but expressed also many concerns about the actual method used and also about the feasibility of such a treatment at a population scale (Curiel and Curiel 2002).

## The missing link

By the end of the 1990s, despite some clear advances related to *in vitro*-transcribed mRNAs, many aspects of the mRNA-based treatment had not been solved. Overcoming the problems related to the ectopic introduction of mRNAs into cells was a piecemeal process. In these years, Karikó and her team first successfully overexpressed a receptor protein in cultured mammalian cells by delivering the encoding mRNA molecules (Karikó et al. 1999). One year later, together with Drew Weissman, she reported that synthetic RNAs are also immunogenic by observing that the introduction of mRNAs encoding antigens to dendritic cells activated potent primary T cell responses *in vitro* (Weissman et al. 2000). The method seemed to be applicable to develop a T cell activating vaccine.

The immunogenicity of the mRNA molecule was still an obstacle for the safe use in humans. After much thinking and experimenting, it was found that among the many types of RNAs, tRNAs did not activate the immune system. Karikó was pondering that the difference could be explained by the much larger amount of modified nucleosides in the tRNA molecules compared to the mRNAs. So, the group set out to introduce non-canonical (modified) nucleosides into their mRNA constructs. Their efforts paid off and in 2005 a comparative investigation revealed that the immunogenic effect can be minimised by the incorporation of such modified nucleosides into the mRNA (Karikó et al. 2005). In addition, mRNA containing pseudouridine produced larger amounts of active protein in injected mice in the absence of immunogenicity (Karikó et al. 2008). Finally, they also showed that HPLC purification of the nucleoside-modified mRNA eliminates the remaining undesirable activation of the immune system (Karikó et al. 2011).

In the following years, several research groups reported the use of mRNA-based vaccination technology in preclinical investigations involving unmodified mRNAs (Kallen et al. 2013; Petsch et al. 2012; Schee et al. 2016). The first meeting on mRNA therapies was organised in Tübingen and the number of participants was much larger than expected. Despite many positive results, Karikó and colleagues remained rather realistic. In a paper, published the same year they wrote, “*the use of mRNA-based therapy in humans is yet to be seen, but our knowledge so far can open new horizons in the development of modern, mRNA-based treatments and bring us ever closer to the realization of their use in the clinic*.” (Boros et al. 2013).

## The finish without an end

The Zika virus outbreak of 2015–16 that occurred in South America resulted in more than 1.5 million infections. By the time of the Olympic Games, organised in Brazil in the summer of 2016, the situation was deemed quite serious. A link between microcephaly of newborns and mothers’ infection during pregnancy was established, and the WHO warned about an extending pandemic across Americas. The absence of specific and approved anti-Zika treatments created a need to develop effective new ones, providing an opportunity for scientists to demonstrate the efficiency and viability of the mRNA technology. Answering this challenge, a year later, Pardi et al. (2017) published an important study in *Nature*, showing that a single low-dose intradermal immunization with lipid-nanoparticle-encapsulated nucleoside-modified mRNA, which encoded the pre-membrane and envelope glycoproteins of a Zika virus strain, could elicit strong and durable neutralizing antibody responses in mice and non-human primates. Clinical trials with the Zika mRNA vaccine also started.

Nevertheless, the real breakthrough occurred three years later, when once again, a previously unknown viral pathogen threatened to create an epidemic of unseen proportions. On the 11th March 2020, the WHO declared a pandemic situation affecting all people world-wide based on the rapid spread of a new coronavirus variant (SARS-CoV-2). No specific cure was available, but the severity of the symptoms associated with the SARS-CoV-2 infections made the fast development of effective treatments an imperative. The sooner an effective vaccine could be developed, the more lives could be saved. Many different strategies were used, and the stage was set for the mRNA-based vaccines to show their power.

## Lessons from the perspective of research and innovation management

The story of Katalin Karikó and the development of mRNA offers many lessons related to the field of research and innovation management. Of these, five will be highlighted: (1) viability of linear innovation models; (2) the role of new technologies; (3) the role of the academic institutions and universities in innovation; (4) perseverance and faith; (5) the role of chance (Rothwell 1994).

### The booster effect of market pull

There are various approaches to understanding the process of innovation, ranging from simple to complex models. Berkhout et al. (2006) classify the development of these models into three generations. The first two are linear models: the Technology Push model, where scientific curiosity is the main driving force, and the Market Pull model, where research is primarily driven by market demands. The third model combines elements from both the Technology Push and Market Pull models, and is well exemplified by the development of mRNA vaccines for COVID-19.

**Fig. 2:**
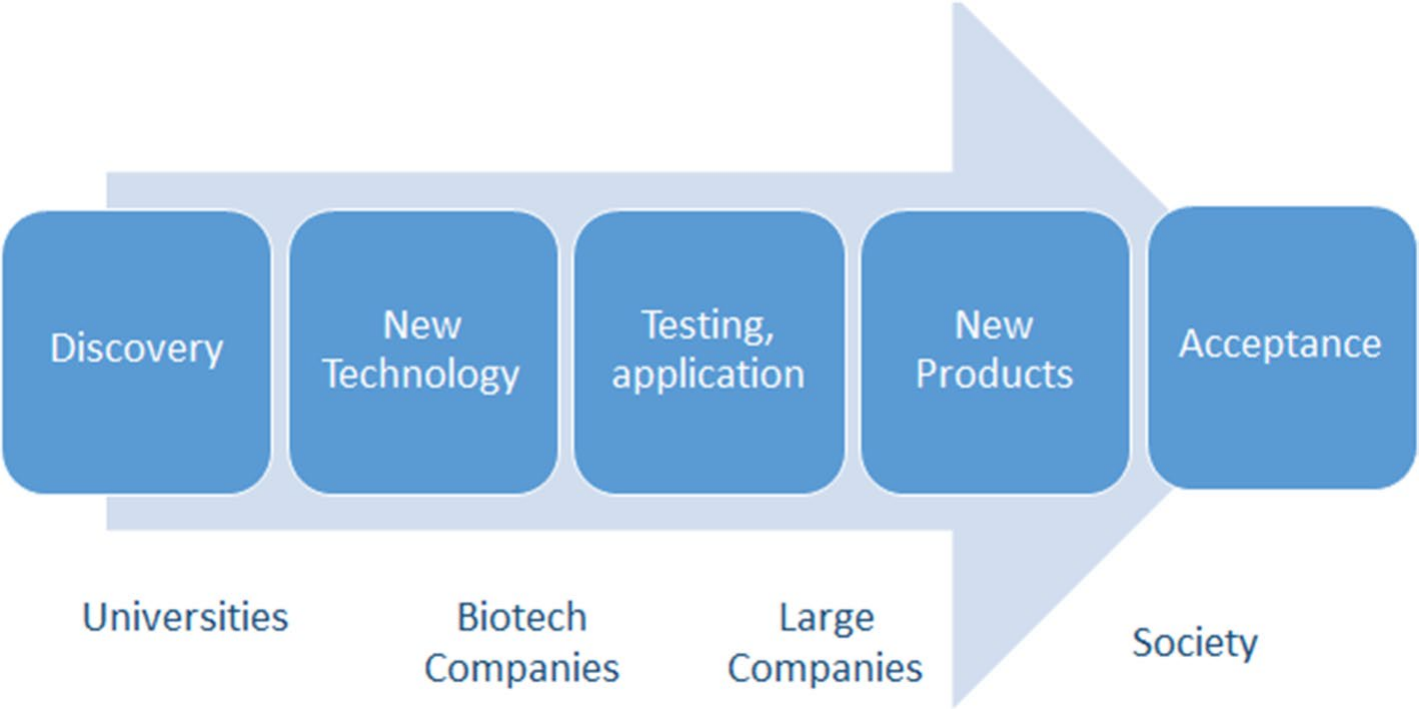
Linear Innovation model fits well to the development of the new vaccination technology based on mRNA

The development of mRNA-based technologies, as demonstrated by Katalin Karikó's model (Fig. 2), sheds light on the interplay between science, technology, and market demand. The process starts with fundamental research, represented by the elements of Discovery and New Technology, followed by applied research such as Testing and Application, leading to development and dissemination of New Products and Adoption.

Market demands had significantly influenced the speed of developments. It is perhaps natural that the crisis and the epidemic triggered the market's pull in relation to product development. Table 1 illustrates how the acceleration of events driven by the needs of markets led to the FDA approval of COVID-19 mRNA vaccines.

**Table 1:** Timeline of rapid development of mRNA vaccines against SARS-CoV-2 in 2020 (based on Barbier et al. 2022)

Date in 2020	Occurrence
12 January	SARS-CoV-2 genomic sequence published
13 January	Moderna: mRNA vaccine sequence designed
12 March	Moderna: first subject doses in phase 1
23 April	*BioNTech-Pfizer*: first subject doses in phases 1–2*
29 May	Moderna: first subject doses in phase 2
27 July	Moderna and *BioNTech-Pfizer*: initiation of phase 3
6 October	Regulatory submissions *BioNTech-Pfizer*: European Medicines Agency
9 October	Regulatory submissions *BioNTech-Pfizer*: Canada
9 November	*BioNTech-Pfizer* publishes interim phase 3 results
16 November	Moderna publishes interim phase 3 results
16 November	Regulatory submissions Moderna: European Medicines Agency
18 November	*BioNTech-Pfizer* publishes phase 3 results
20 November	Regulatory submissions *BioNTech-Pfizer*: USA
30 November	Regulatory submissions Moderna: USA
30 November	Moderna publishes phase 3 results
11 December	*BioNTech*162b2 receives EUA in USA
18 December	Moderna mRNA-1273 receives EUA in USA

The table also shows that the two large pharmaceutical companies are competing not only with time but also with each other

*BNT162 phase 1–2 trial investigated several drug candidates, with BNT162b2 selected for phase 3 trials

The development and market entry of mRNA technology shows that, in addition to the decades-long research-driven Technology Push, a sudden market pull radically accelerates the innovation process.

Market demand has played a significant role in accelerating the pace of developments, particularly during times of crisis and epidemics. Table 1 highlights how the urgent need for a COVID-19 vaccine drove market demand and led to the FDA's approval of mRNA vaccines.

The success of mRNA technology in entering the market illustrates that innovation can be rapidly accelerated not only through decades-long research-driven Technology Push, but also by sudden market pull.

### The role of new technologies

Biological research technologies arise in a variety of different ways from inspired insight to incremental advances related to engineering development even by serendipity. Technologies may emerge in a completely unpredictable and unplanned fashion (Fields 2001).

Developments in key enable technologies drive innovation throughout the economy and affect various industries. Key enable technologies are characterized by rapid innovation cycles, high levels of research and development (R & D), significant human and infrastructural investment, and significant digital support. Katalin Karikó’s career highlights this path of innovation following new emerging biotechnologies typical of today (e.g., within genome editing technology, CRISPR-Cas9, TALEN, Zinc Finger Nucleases Cell, within gene therapies CAR T cells—Kymriah and AAV gene therapy—Luxturna).

RNA technologies, particularly mRNA and siRNA, have emerged as key drivers of innovation not only in the healthcare industry but also in other sectors such as agriculture and the food industry. The rapid pace of technological advancements has led to shorter product cycles and the blurring of industry boundaries, a phenomenon referred to by Kodama (1985) as the "*technological fusion process.*" The synergy between technology and biology continues to spur innovation, and it is crucial to have adequate funding and institutional support to bridge the gap between research and practical applications. This will enable the full utilization of research results and the realization of the potential benefits of RNA technologies across diverse industries.

### The role of academic institutions in innovation

The crucial role of universities and academic institutions in successful research cannot be overstated. Katalin Karikó and her co-authors' ground-breaking work at the University of Pennsylvania resulted in the development of mRNA vaccines, which have been critical in combating the COVID-19 pandemic. While academic institutions play an essential role in research, their ability to bring research results to market varies significantly. Some universities excel in this area, while others struggle. To achieve successful research outcomes, institutions must prioritize openness, foster excellent internal communication, and create larger research units. Additionally, reaching a critical mass of researchers and providing infrastructural support are vital for achieving technological development that can spark industry or societal interest.

For modern universities to thrive, a strong commitment to research and development (R&D) is essential. Research plays a crucial role in the innovation process, but the time and costs involved can be difficult to estimate at the outset. Universities must provide a conducive environment for R&D activities, including suitable working conditions and an effective monitoring system to track progress. The research process can be lengthy, spanning several years or even decades, and may not always result in a tangible product. Katalin Karikó's ground-breaking research, for example, took several decades to come to fruition. However, the urgent need for solutions during the COVID-19 pandemic propelled the rapid utilization of her research results in the development of novel vaccines, which were quickly made available to the public.

The Pfizer/BioNTech and Moderna COVID-19 mRNA vaccines are based on licensed knowledge from the University of Pennsylvania. As a result of this licensing agreement, the university has received significant financial benefits from the sale of these products and continues to do so (Penn Medicine News 2020).

### Perseverance and faith

Achieving success in innovation often demands significant dedication, unwavering faith, and years of persistent work. However, securing investment for innovative ideas is not always easy or predictable. Inventors and researchers often assume that patenting their research results is sufficient to attract investors who will handle the remaining innovation tasks. However, this is not always the case, and additional effort may be required to secure funding and support for innovative projects.

Despite facing years of research setbacks and recognition failures, Katalin Karikó and her team persisted in their work on mRNA-induced inflammation. In 2005, their persistence paid off with a ground-breaking breakthrough. However, their findings were initially underappreciated, and several leading medical journals rejected their report before it was finally published in Immunity that same year (Karikó et al. 2005). Although the researchers anticipated significant interest from the scientific community and industry, it did not materialize immediately. Nonetheless, they patented their discoveries and launched RNARx in 2006, a company focused on developing mRNA therapies for a variety of diseases. Unfortunately, the company was short-lived and ultimately closed due to financial difficulties. It was not until five years after the publication of their results that their discovery caught the attention of two biotech startups: Moderna Inc in Cambridge, MA, US, and BioNTech Gmbh in Mainz, Germany. Both companies eventually licensed the Karikó-Weissman patent, leading to the creation of the Pfizer/BioNTech and Moderna COVID-19 mRNA vaccines (Yu 2021).

### The role of chance

Throughout history, accidental or unexpected innovations have often played a crucial role in driving progress. The encounter between Katalin Karikó and Drew Weissman, for example, was entirely serendipitous. While making copies of an article in a research journal, Karikó struck up a conversation with Weissman, leading the two scientists to discover their shared interest in mRNA. Weissman later remarked, "*I've always wanted to try mRNA and here was someone at the Xerox machine who said she was doing it*" (Yu 2021).

Even in well-planned R&D projects, unexpected events can sometimes lead to innovative breakthroughs. While they may be initially disruptive or inconvenient, these situations can lead to new directions for development. For instance, the active ingredient in Viagra was initially intended for heart medicine, while a chemist's mixture of substances led to the invention of Post-it notes, a glue with weak adhesion properties (Goldstein et al. 2019; Karapapa 2019).

Serendipitous discoveries often arise unexpectedly and may lead to significant breakthroughs. However, the term serendipity can be controversial as it suggests that discoveries happen purely by chance. In reality, serendipitous discoveries often arise from a combination of preparation, opportunity, and creativity. Researchers must be prepared to recognize and capitalize on unexpected results, and the research environment should encourage exploration and risk-taking. As Louis Pasteur once said, "*chance favours the prepared mind*." Thus, researchers should view coincidences as a potential source of ideas only if they are equipped with the knowledge, skills, and resources to recognize and develop them (Deák 2021).

## Conclusion for future biology

The success of mRNA vaccines has generated significant excitement about the potential for this technology to transform medicine. Researchers are actively pursuing the development of vaccines for diseases such as Zika, HIV, malaria, tuberculosis, and cancer. However, the path forward is likely to be fraught with challenges that are yet unknown. Nonetheless, even if only a fraction of these hopes comes to fruition, the success of mRNA vaccines demonstrates how collaboration and regulated competition can lead to rapid and positive outcomes. It would be highly advantageous for all players in the field, including universities, small and medium-sized enterprises, and regulatory bodies, to draw lessons from this unique event and work towards creating an improved ecosystem that facilitates the transformation of research ideas into reality.
